# Aerosol delivery with two ventilation modes during mechanical ventilation: a randomized study

**DOI:** 10.1186/s13613-016-0169-x

**Published:** 2016-07-22

**Authors:** Jonathan Dugernier, Gregory Reychler, Xavier Wittebole, Jean Roeseler, Virginie Depoortere, Thierry Sottiaux, Jean-Bernard Michotte, Rita Vanbever, Thierry Dugernier, Pierre Goffette, Marie-Agnes Docquier, Christian Raftopoulos, Philippe Hantson, François Jamar, Pierre-François Laterre

**Affiliations:** Soins Intensifs, Médecine Physique, Cliniques universitaires Saint-Luc, Avenue Hippocrate 10, 1200 Brussels, Belgium; Médecine Physique, Cliniques universitaires Saint-Luc, Avenue Hippocrate 10, 1200 Brussels, Belgium; Institut de Recherche Expérimentale et Clinique (IREC), Pneumologie, ORL & Dermatologie, Université catholique de Louvain, 1200 Brussels, Belgium; Soins Intensifs, Cliniques universitaires Saint-Luc, Avenue Hippocrate 10, 1200 Brussels, Belgium; Médecine Nucléaire, Cliniques universitaires Saint-Luc, Avenue Hippocrate 10, 1200 Brussels, Belgium; Soins Intensifs, Clinique Notre-Dame de Grâce, Chaussée de Nivelles 212, Gosselies, Belgium; Haute Ecole de Santé Vaud, Filière physiothérapie, University of Applied Sciences and Arts Western Switzerland, Avenue de Beaumont 21, 1011 Lausanne, Switzerland; Louvain Drug Research Institute (LDRI), Université catholique de Louvain, Avenue Hippocrate 10, 1200 Brussels, Belgium; Soins Intensifs, Clinique Saint-Pierre, Avenue Reine Fabiola 9, 1340 Ottignies, Belgium; Radiologie Interventionnelle, Cliniques universitaires Saint-Luc, Avenue Hippocrate 10, 1200 Brussels, Belgium; Anesthésiologie, Cliniques universitaires Saint-Luc, Avenue Hippocrate 10, 1200 Brussels, Belgium; Neurochirurgie, Cliniques universitaires Saint-Luc, Avenue Hippocrate 10, 1200 Brussels, Belgium

**Keywords:** Aerosol delivery, Ventilation mode, Invasive mechanical ventilation, Vibrating-mesh nebulizer

## Abstract

**Background:**

Volume-controlled ventilation has been suggested to optimize lung deposition during nebulization although promoting spontaneous ventilation is targeted to avoid ventilator-induced diaphragmatic dysfunction. Comparing topographic aerosol lung deposition during volume-controlled ventilation and spontaneous ventilation in pressure support has never been performed. The aim of this study was to compare lung deposition of a radiolabeled aerosol generated with a vibrating-mesh nebulizer during invasive mechanical ventilation, with two modes: pressure support ventilation and volume-controlled ventilation.

**Methods:**

Seventeen postoperative neurosurgery patients without pulmonary disease were randomly ventilated in pressure support or volume-controlled ventilation. Diethylenetriaminepentaacetic acid labeled with technetium-99m (2 mCi/3 mL) was administrated using a vibrating-mesh nebulizer (Aerogen Solo^®^, provided by Aerogen Ltd, Galway, Ireland) connected to the endotracheal tube. Pulmonary and extrapulmonary particles deposition was analyzed using planar scintigraphy.

**Results:**

Lung deposition was 10.5 ± 3.0 and 15.1 ± 5.0 % of the nominal dose during pressure support and volume-controlled ventilation, respectively (*p* < 0.05). Higher endotracheal tube and tracheal deposition was observed during pressure support ventilation (27.4 ± 6.6 vs. 20.7 ± 6.0 %, *p* < 0.05). A similar penetration index was observed for the right (*p* = 0.210) and the left lung (*p* = 0.211) with both ventilation modes. A high intersubject variability of lung deposition was observed with both modes regarding lung doses, aerosol penetration and distribution between the right and the left lung.

**Conclusions:**

In the specific conditions of the study, volume-controlled ventilation was associated with higher lung deposition of nebulized particles as compared to pressure support ventilation. The clinical benefit of this effect warrants further studies.

*Clinical trial registration* NCT01879488

**Electronic supplementary material:**

The online version of this article (doi:10.1186/s13613-016-0169-x) contains supplementary material, which is available to authorized users.

## Background

Combining aerosol therapy with invasive mechanical ventilation is common in the intensive care unit (ICU) [1].

In vitro and experimental studies have been performed to optimize aerosol lung deposition [2, 3]. Many factors related to the device, the artificial airways and the respiratory pattern inherent to invasive mechanical ventilation influence lung deposition [4]. Most of them should be controlled to deliver high doses of concentration-dependent drugs such as antibiotics. Vibrating-mesh nebulizers ensure currently the best drug output, ergonomy and practical use in the ICU [5, 6]. Applying a constant inspiratory flow pattern with a slow inspiratory flow rate improved aerosol delivery as compared to a decelerating inspiratory flow pattern with a higher peak inspiratory flow rate [7]. This mandates volume-controlled ventilation modality and a deep depression of the respiratory drive with sedatives [8]. Conversely, reducing sedation is promoted in ICU in order to preserve the diaphragmatic function through a spontaneous breathing activity and hence to shorten the duration of mechanical ventilation [9, 10]. The decelerating inspiratory flow pattern and the spontaneous breathing activity of the patient (uncontrolled respiratory rate, inspiratory flow rate) related to the pressure support modality may affect aerosol delivery to the lungs. The comparison of pressure support ventilation and volume-controlled ventilation regarding aerosol administration has not been investigated.

Recent guidelines focused on the standardization of radionuclide imaging methods validated to assess lung deposition [11]. Imaging techniques are able to highlight the aerosol distribution into the lungs [12]. Twenty years ago, scintigraphic studies reported a low aerosol lung deposition during invasive mechanical ventilation with a jet and ultrasonic nebulizer [13–16]. Since then, pharmacokinetic studies showed high concentrations of inhaled antibiotics through the lungs after dissection of subpleural lung segments in piglets [17] and after endotracheal suctioning [18] or bronchoalveolar lavage [19, 20] in patients suffering from ventilator-associated pneumonia. However, topographic assessment of lung deposition by imaging technique during invasive mechanical ventilation has not been investigated.

The aim of this study was to compare in vivo the impact of two ventilation modalities, pressure support versus volume-controlled ventilation, on lung dose of a radiolabeled aerosol administered with a vibrating-mesh nebulizer during invasive mechanical ventilation.

## Methods

### Study design and patient selection

This randomized, comparative, double-blind study included postoperative neurosurgery ventilated patients with a healthy lung function. Eligibility criteria were aged 18 years and older, admitted for brain neurosurgery and the availability of krypton gas (^81m^Kr). Patients were included if the FEV1 to FVC ratio was superior to 70 %. Exclusion criteria were spine neurosurgery, history of cardiovascular and pulmonary disease, extubation immediately after surgery, the allocated ventilation mode modification during the nebulization and inaccurate quantification of aerosol deposition (artifacts or overlap of tracheal and pulmonary deposition).

The study protocol was approved by the Institutional Medical Ethics Committee (B403201317342). Written informed consent was obtained from all participants. Preoperative pulmonary function testing was performed in the Neurosurgery Department according to the American Thoracic Society guidelines using a MicroLoop spirometer (CareFusion, San Diego, CA) [21]. The two procedures were randomized by a computer-generated random number list. Generation of the random allocation sequence, enrollment of the patients and allocation to the assigned ventilation mode were performed by the same clinician author (JD). The double-blind design was related to the patients and the data analysis. The nebulizer procedure and image acquisition were performed in the same room in the Nuclear Medicine Department.

### Invasive mechanical ventilation and nebulization procedure

**Fig. 1:**
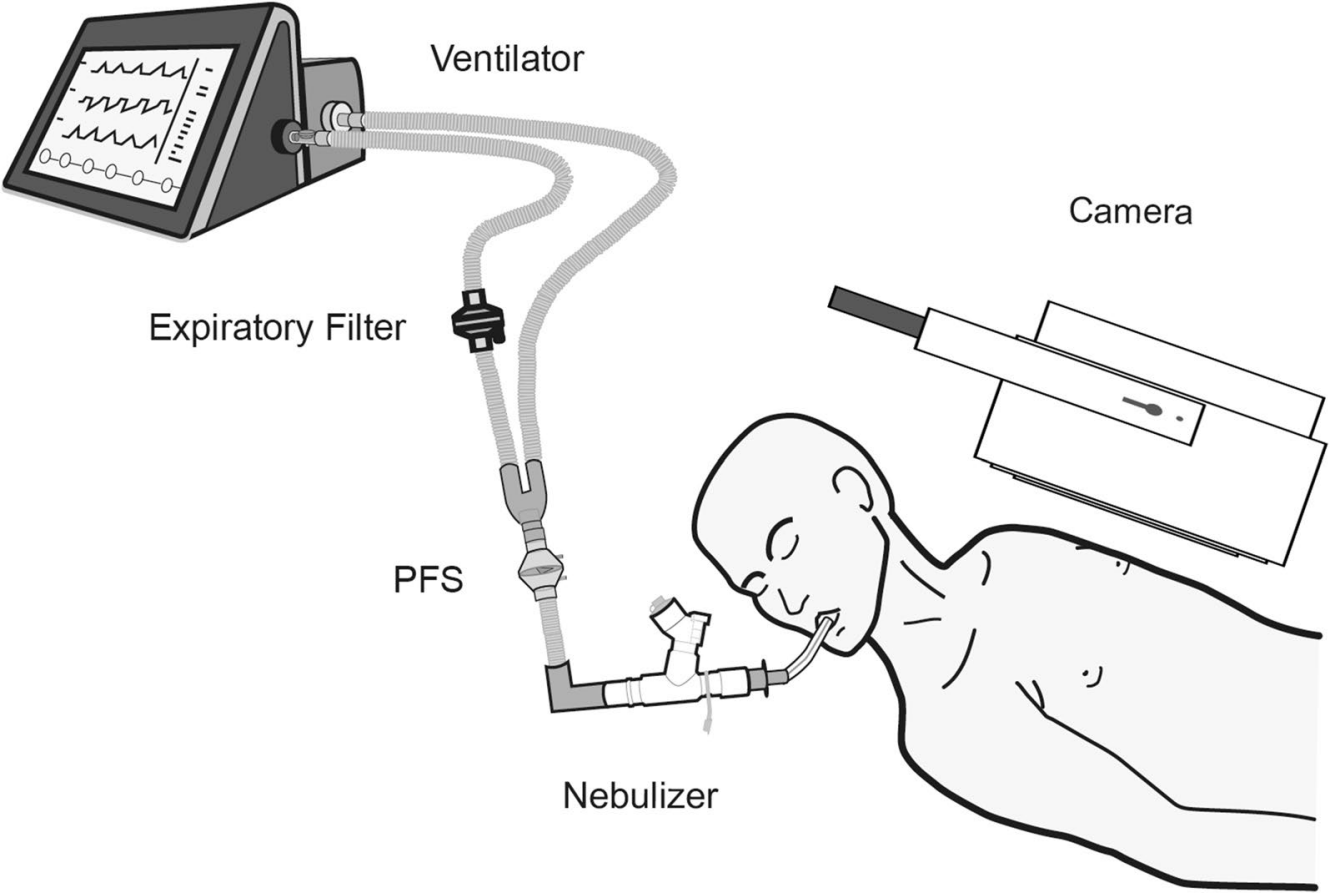
Planar imaging to assess aerosol lung deposition during invasive mechanical ventilation. The ventilator was equipped with a 160-cm, 22-mm inner-diameter ventilator circuit (IMMED, Bruxelles, Belgium) including a 7-cm proximal flow sensor (PFS, Hamilton Medical, Bonaduz, Switzerland) positioned between the Y-piece and the catheter mount, a protection filter and the vibrating-mesh nebulizer. The patient was in semirecumbent position at 15° with the head turned *right* to avoid the overlap of the thorax, the ventilator circuit and the gamma camera (at 10 cm of the sternum)

Patients were randomly assigned to pressure support (PSV) or volume control (VCV) mode. Patients were ventilated using an ICU turbine-driven ventilator (Bellavista 1000e, Imtmedical, Buchs, Switzerland) (Fig. 1). Aerosol particles were generated continuously by a vibrating-mesh nebulizer (Aerogen Solo^®^, provided by Aerogen Ltd, Galway, Ireland). The nebulizer was placed between the endotracheal tube and the catheter mount because of the presence of a proximal flow sensor. The nebulizer reservoir was filled with technetium-99m-labeled diethylenetriaminepentaacetic acid (^99m^Tc-DTPA, 2 mCi/3 mL).

The sedative drug (propofol) was titrated in both groups to avoid patient’s movements, cough and patient-ventilator asynchrony during the procedure, while keeping an exclusive spontaneous breathing activity during PSV. Patients in VCV did not trigger the ventilator based on the absence of spontaneous respiratory rate as assessed by continuous observation of the ventilator tracings during the procedure. The ventilatory pattern during VCV was: tidal volume of 8 mL/kg and respiratory rate targeting a minute ventilation around 8 L/min during the postoperative awakening phase. Inspiratory time and respiratory rate were then adjusted to ensure an inspiratory/expiratory ratio of 30 % with a constant inspiratory flow of 30 L/min. As there is no end-inspiratory pause in PSV, none was imposed in VCV.

The inspiratory pressure level in PSV was set to reach a tidal volume of 8 mL/kg, and the expiratory trigger was set to obtain an inspiratory/expiratory ratio of 30 %. Positive end-expiratory pressure was set at 5 cmH_2_O for all patients. A bias flow of 10 L/min was imposed by the ventilator. Neither heating nor humidification systems were used to condition inspired air but a heat and moisture exchanger (HME) filter that was removed during the nebulization as suggested previously [8]. A HME filter was placed on the expiratory limb during nebulization to measure the exhaled dose and to avoid radioactive particles liberation.

### Particle size analysis

Particle size analysis of the radiolabeled aerosol was assessed for a decelerating (peak inspiratory flow of 60 L/min) and a constant inspiratory flow pattern (30 L/min) that characterizes the PSV and VCV mode. We used an eight-stage Andersen Cascade Impactor (Copley Scientific Ltd, Nottingham, UK) based on pharmacopeia and according to the bench model described by Miller et al. [22] (Additional file 1). The stage at the median amount of radioactivity defined the mass median aerodynamic diameter (MMAD).

### Image acquisition and deposition analysis

**Fig. 2:**
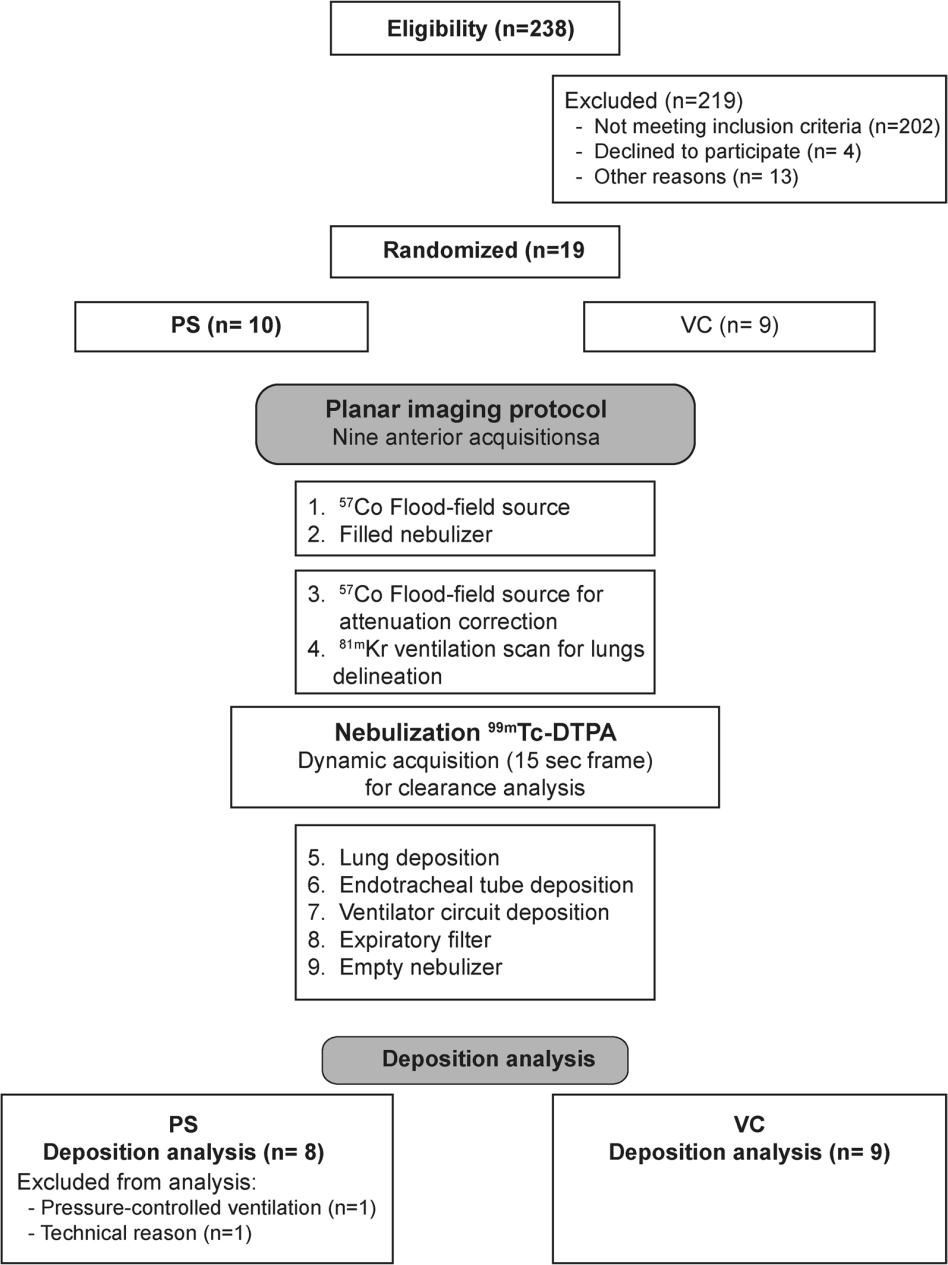
Summary of the protocol

Image acquisitions were performed using a planar single detector gamma camera (STARPORT 400 AC/T, GE, Hørsholm, Denmark). Nine static anterior acquisitions of 2 min were required for counts correction for background, decay and attenuation and to analyze the pulmonary and extrapulmonary deposition according to recent international recommendations (Fig. 2) [23, 24]. Delineation of lung outlines, regions of interest and counts quantification were performed using the Odyssey program (LR Software (v7.0-1.7), Philips).

All data were anonymized by a code number. Data analysis was blinded, i.e., performed by a physician of the Nuclear Medicine Department without knowledge neither of the ventilation mode used nor of any particular clinical parameters.

Right and left lung deposition was separately measured using a rectangular ROI dimensioned on lung outlines from the 15 % isocount contour of the ^81m^Kr radioactive gas scan [23]. Whole lung deposition was derived from the quantification of counts included in these both ROIs. Counts correction for background, decay and attenuation was performed as described in previous studies [23–25] (Additional file 1). A right to the left lung deposition ratio was calculated to compare both lung depositions. A penetration index defined the penetration of aerosol particles from an inner to an outer rectangular lung region designed from the ^81m^Kr ventilation scan and reported on the ^99m^Tc-DTPA scan. The penetration index was calculated as the outer to inner lung region ratio (O/I) from the ^99m^Tc-DTPA acquisition normalized to the O/I ratio from the ^81m^Kr gas acquisition as described previously [23]. Extrapulmonary deposition was determined by the total count measured in the nebulizer reservoir minus whole lung count. Deposition analysis within the endotracheal tube and the trachea was determined by the total extrapulmonary deposition minus the total count in the ventilator circuit and retained in the expiratory filter and the nebulizer reservoir. It was grouped due to the difficult differentiation of the deposition in the distal part of the endotracheal tube and the trachea. Radiolabeled deposition on each component of the ventilator circuit and the expiratory filter was quantified using a ROI fitted to their size. According to the ventilator circuit, the term “proximal” was used when the pieces were close to the patient, i.e., the proximal flow sensor, the catheter mount and the nebulizer T-piece.

Pulmonary deposition and extrapulmonary deposition of radiolabeled particles were expressed in counts and as a percentage of the nominal dose (i.e., the amount of radioactivity placed in the nebulizer at the beginning of experiments).

### Statistical analysis

Statistical analysis was performed using SPSS software (version 20.0, IBM software). Sample size calculation was based on a 7 % expected difference in mean deposition for a statistical power of 80 % [3, 26]. Data are expressed as mean ± standard deviation (SD) or median (25–75 % interquartile range (IQR)) depending on the normality tested using the Kolmogorov–Smirnov test. Comparison of both ventilation modes was conducted with independent Student *t* tests or Mann–Whitney test. Both lungs were compared using a Wilcoxon test for paired organs. Correlation between aerosol lung deposition and the ventilatory pattern was conducted using the Pearson correlation coefficient (*r*). The intersubject variability of whole lung deposition was determined by the calculation of the coefficient of variation (CV). A *p* value lower than 0.05 was considered significant.

## Results

Two hundred thirty-eight patients schedule for postoperative ICU admission were screened for eligibility between July 2013 and June 2015 (completion date according to the sample size calculation). Among nineteen randomized patients, two patients allocated to PSV were excluded. One patient was excluded because mandatory sedation during the procedure implied to switch from PSV to pressure-controlled ventilation. The other patient was excluded because quantification of aerosol lung deposition was impossible due to technical reasons. The study included seventeen postoperative neurosurgery ventilated patients, eight in PSV and nine in VCV (Fig. 2). Patient characteristics are depicted in Table 1.

**Table 1:** Patient characteristics

	PSV (*n* = 8)	VCV (*n* = 9)	*p* value
Age (years)	56 ± 8	61 ± 11	0.254
Male, *n* (%)	5 (62.5)	4 (44.4)	
Height (cm)	169 ± 10	165 ± 11	0.441
Body weight (kg)	74 ± 14	68 ± 21	0.431
Ideal body weight (kg)	65 ± 10	59 ± 10	0.516
Smoker, *n* (%)	3 (37.5)	3 (33.3)	
Surgery, *n* (%)			
Brain tumor resection	3 (37.5)	2 (22.2)	
Embolization of intracranial unruptured aneurysm	3 (37.5)	0	
Neurosurgical clipping of a unruptured intracranial aneurysms	1 (12.5)	6 (66.7)	
Stereotactic brain biopsy	1 (12.5)	0	
Vestibular schwannoma resection	0	1 (11.1)	
Lung function			
FEV_1_ (% predicted value)	95 ± 16	97 ± 9	0.777
FVC (% predicted value)	100 ± 19	101 ± 14	0.927
FEV_1_/FVC	77 ± 5	79 ± 6	0.509

Quantitative variables are expressed as mean ± SD. Qualitative variables are expressed as a proportion (%)

*FEV*_*1*_ forced expiratory volume in 1 s, *FVC* forced vital capacity

Nebulization lasted on average 9 ± 3 min. The dynamic acquisition revealed a linear increase in counts in lung ROI that confirmed the absence of clearance of the aerosol during nebulization. The absence of potential radioactivity quantified on the expiratory valve of the ventilator ensured the effective retention of exhaled particles in the HME filter. No ambient and surface contamination was detected after the procedure.

The MMAD at the distal tip of the endotracheal tube was 2.1 ± 0.07 and 1.95 ± 0.37 µm with a constant inspiratory flow pattern of 30 L/min and a decelerating inspiratory flow pattern with a peak inspiratory flow of 60 L/min, respectively. Mechanical ventilation settings and ventilatory pattern during inhalation are presented in Table 2. A similar inspiratory time was observed with both ventilation modes. The decelerating inspiratory flow pattern during PSV resulted in a higher inspiratory flow rate. Patients had a lower respiratory rate during PSV, resulting in a longer expiratory time.

**Table 2:** Mechanical ventilation details and ventilatory pattern during inhalation

	PSV (*n* = 8)	VCV (*n* = 9)	*p* value
Sedatives (propofol, mg/h)	190 (160–252)	200 (160–260)	0.622
ETT diameter (mm)	8.0 (7.5–9.0)	7.0 (7.5–8.5)	0.252
^81m^Kr ventilation distribution			
Right/left lung ratio	1.13 ± 0.25	1.09 ± 0.32	0.755
Ventilatory pattern during inhalation			
*P*_peak_ (cmH_2_O)	16 ± 3	20 ± 1	*0.005*
*V*_T insp_ (mL)	586 ± 117	530 ± 95	0.292
*V*_T insp_ (mL/kg IBW)	8.65 (8.00–8.90)	8.70 (8.55–9.20)	0.439
RR (cycle/min)	14 ± 1	18 ± 2	*<0.001*
MV_insp_ (L/min)	8.01 ± 1.48	9.17 ± 0.92	0.070
Flow_peak insp_ (L/min)	44 ± 8	32 ± 4	*0.002*
*T*_insp_ (s)	1.15 ± 0.19	1.12 ± 0.12	0.664
*T*_exp_ (s)	3.72 (2.91–3.99)	2.24 (2.16–2.55)	*0.001*
*T*_insp_ *T*_Tot_ (%)	25 (22–34)	32 (32, 33)	0.072

Data expressed as mean ± SD or median (25–75 % IQR)

*P*-value in italic is considered significant (*p* < 0.05)

*ETT* endotracheal tube, *MV* minute ventilation, *P* pressure, *RR* respiratory rate, *T*_*insp*_ inspiratory time, *T*_*insp*_ *T*_*Tot*_ inspiratory time to breathing cycle time ratio, *T*_*exp*_ expiratory time, *V*_*T*_ tidal volume

Pulmonary and extrapulmonary deposition and the penetration index according to both ventilation modes are detailed in Table 3. The attenuation correction factor was 1.65 ± 0.19 and 1.56 ± 0.18 in PSV and VCV, respectively. The deposited drug amount into the lungs was significantly higher during VCV (*p* = 0.038), while a higher aerosol deposition on the endotracheal tube, the trachea and main bronchi was observed during PSV (*p* = 0.043). We observed a similar aerosol deposition between both ventilation modes in each lung analyzed separately (*p* = 0.057 and *p* = 0.885 for the right and the left lung, respectively). The penetration index was also comparable between both modes for the right (*p* = 0.210) and the left lung (*p* = 0.211).

**Table 3:** Aerosol deposition in seventeen postoperative neurological patients

	PSV (*n* = 8)	VCV (*n* = 9)	*p* value
*Pulmonary deposition (%)*	*10.5* *±* *3.0 (28)*	*15.1* *±* *5.0 (33)*	*0.038*
Right lung	6.1 ± 1.9 (31)	10.6 ± 5.8 (55)	0.057
Penetration index	0.75 (0.30–0.94)	0.32 (0.16–0.77)	0.210
Left lung	4.1 (3.8–4.6)	4.5 (2.2–5.6)	0.885
Penetration index	0.67 (0.53–0.86)	0.74 (0.6–1.06)	0.211
Right/left lung ratio	1.39 (0.91–2.05)	3.33 (0.7–5.38)	0.336
*Extrapulmonary deposition (%)*	*89.5* *±* *3.0*	*84.9* *±* *5.0*	*0.038*
ETT and tracheal area	27.4 ± 6.6 (24)	20.7 ± 6.0 (29)	*0.043*
Expiratory filter	23.7 ± 5.3 (22)	22.5 ± 7.6 (34)	0.710
Ventilator circuit	34.7 ± 8.7 (25)	38.4 ± 12.3 (32)	0.486
Proximal pieces	32.0 ± 7.4 (23)	35.9 ± 12.5 (35)	0.451
Insp–expi tubing	2.7 ± 1.9 (70)	2.5 ± 1.7 (68)	0.833
Nebulizer retention	3.7 ± 0.9 (24)	3.3 ± 0.7 (21)	0.334

Data expressed as mean ± SD (coefficient of variation, %) or median (25–75 % IQR). Proximal pieces of the ventilator circuit included the catheter mount, the nebulizer T-piece and the proximal flow sensor

*P*-value in italic is considered significant (*p* < 0.05)

*ETT* endotracheal tube

If we compare aerosol deposition between both lungs, there was no significant difference of deposition between the right and the left lung (*p* = 0.093 and *p* = 0.066 during PSV and VCV, respectively). The penetration index of the right and the left lung was comparable during PSV (*p* = 1.000) and during VCV (*p* = 0.066).

**Fig. 3:**
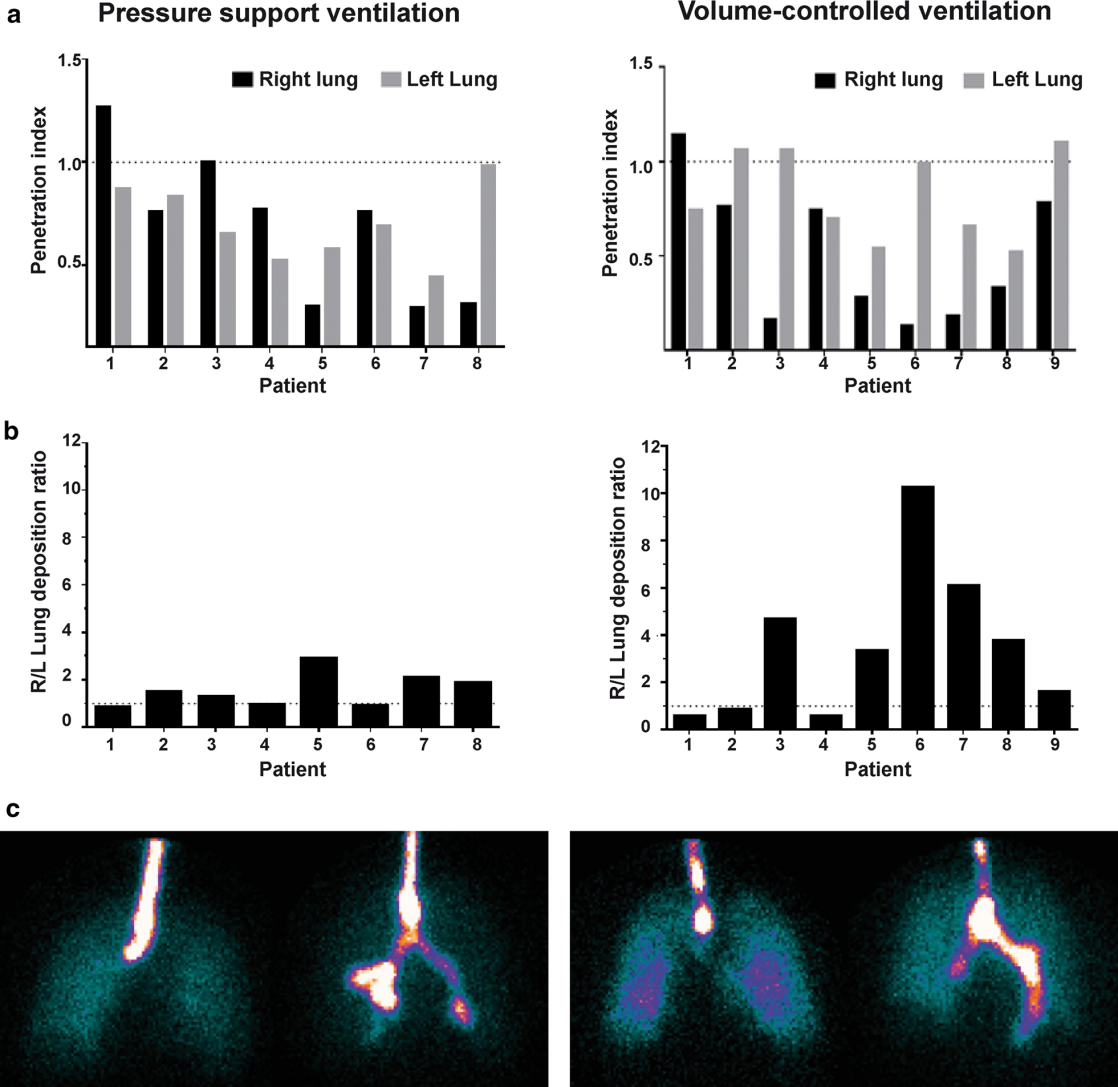
High intersubject variability of aerosol penetration through the lungs and its deposition between the right and the left lung during pressure support ventilation and volume-controlled ventilation. **a** A penetration index equal to 1 indicated a linear aerosol penetration from the inner to the outer part of the lungs. Particles deposition was limited to the central airways with both ventilation modes. **b** A right/left lung deposition ratio equal to 1 indicated a similar aerosol deposition in both lungs. Right lung deposition was predominant with both ventilation modes, especially during volume-controlled ventilation. **c** Scintigraphic images of aerosol lung deposition in two patients in volume-controlled ventilation (*left*) and two patients in pressure support ventilation (*right*). With both ventilation modes, the first patient on the left benefits of a symmetrical aerosol lung deposition while a predominant left lung or right lung deposition is depicted in the patient on the right. Aerosol penetration from the inner to the outer lung region varies also among patients

Pulmonary aerosol deposition was highly variable among patients with a coefficient of variation of 28 % and 33 % during PSV and VCV, respectively. A high variability of the penetration index and the right to the left lung deposition ratio was also found with the two ventilation modes (Fig. 3a, b). Intersubject variability of aerosol lung deposition whatever the ventilation mode is illustrated in Fig. 3c.

No correlation between lung deposition and the inspiratory flow rate was found for the two modes. However, lung deposition was correlated with the respiratory rate (*r* = 0.741, *p* = 0.036) and inversely correlated with the expiratory time (*r* = −0.846, *p* = 0.008) during PSV.

Extrapulmonary aerosol deposition was higher during PSV (*p* = 0.038) due to a higher deposition on the endotracheal tube, the trachea and main bronchi. A similar aerosol deposition on the ventilator circuit (*p* = 0.486) and the expiratory filter (*p* = 0.710) was observed with both ventilation modes. Around 3 % of the nominal dose was retained on the nebulizer reservoir at the end of nebulization.

## Discussion

This study showed that volume-controlled ventilation increased aerosol delivery to the lungs as compared to pressure support ventilation with a vibrating-mesh nebulizer connected to the endotracheal tube. Lung dose and the site of deposition were highly variable among patients with both ventilation modes.

This study demonstrated better aerosol delivery to the lungs (between 10.5 and 15.1 % of the nominal dose) through an endotracheal tube as compared to previous scintigraphic studies (2.1–5.3 % of the nominal dose) [13, 14, 16]. High residual volume retained in the reservoir (29.6–51.5 % of the nominal dose) of their jet and ultrasonic nebulizers, delayed actuation of their breath-actuated jet nebulizer and deposition analysis performed on patients with respiratory failure (ARDS, COPD, neuromuscular disorders, open-heart surgery) were factors pointed out by previous authors that affected lung delivery and hence explain this discrepancy [13, 14, 16].

Lung deposition of 15 % reported during volume-controlled ventilation is consistent with the amount of amikacin measured in vitro at the distal tip of the endotracheal tube using the same nebulizer position and ventilatory settings [7]. However, lung deposition is still low in comparison with other studies using breath-actuated jet nebulizers (15.3 ± 9.5 % of radiotracer deposited in the lungs) [15], continuous vibrating-mesh nebulizers (40–60 % of antibiotics deposited in piglets lungs) [26, 27] and a recent breath-actuated vibrating-mesh nebulizer connected to the endotracheal tube (72 ± 11 % of aerosol delivery to a lung model) [20]. The explanation is the nebulizer configuration used in the present study, i.e., connected to the endotracheal tube for a continuous nebulization. It led to a significant aerosol loss during the expiratory time [5]. A more distal position was used in other studies using continuous nebulization (from 30 cm of the Y-piece to the inspiratory inlet of the ventilator) [3, 27]. At the time of the study, the proximal flow sensor was considered as a potential barrier for nebulized particles, implying to position the nebulizer after this sensor just before the endotracheal tube. However, we showed recently that the proximal flow sensor has no impact when the nebulizer is placed at 45 cm of the Y-piece or connected to the inspiratory inlet of the ventilator whatever the inspiratory flow pattern (decelerating or constant) [7]. Both positions could have led to a better aerosol delivery with both ventilation modes and should be assessed in further in vivo studies.

Many factors may explain the lower lung deposition during pressure support. The lower respiratory rate (while keeping a similar inspiratory time and tidal volume) during pressure support led to a decrease in lung deposition as compared to volume control. Reducing the respiratory rate without increasing neither the inspiratory time nor the tidal volume led to an increase in the expiratory time and hence reduced aerosol delivery distal to the endotracheal tube during continuous nebulization [28]. Aerosol lung deposition normalized to the respiratory rate becomes similar to the two modes (*p* = 0.378). The low respiratory rate in pressure support was due to the minimal sedation mandatory to the safe postoperative management of neurosurgery patients. Less patient sedation may lead to a higher respiratory rate and hence better lung deposition during pressure support providing that there is no increase of the inspiratory flow rate.

The higher inspiratory flow rate during pressure support ventilation could also explain the lower lung deposition with this mode. It is well known that the higher the inspiratory flow rate, the lower the lung deposition of aerosolized particles [29, 30]. However, lung deposition was independent of the inspiratory flow rate variation between the two modes tested in this study.

The aerosol penetration from the inner to the outer part of the lungs was similar to the two modes. One major factor that influences aerosol penetration is the particle size [31]. A comparable MMAD around 2 µm distal to the endotracheal tube was obtained whatever the inspiratory flow pattern.

As reported by Thomas et al. [16], we found a penetration index around 0.5 for both lungs. A penetration index below 1 indicates a more central deposition of aerosol [32, 33]. Poor aerosol penetration during invasive mechanical ventilation could be explained by particles impaction and trickling from the endotracheal tube to the trachea and main bronchi [14, 16, 34].

The high intersubject variability of the aerosol lung dose, lung penetration and the heterogeneous regional distribution whatever the ventilation mode in patients with healthy lungs is the major finding of this study. We expected that the uncontrolled ventilatory pattern during pressure support ventilation would induce a higher variability of aerosol lung deposition. Surprisingly, a higher variability of lung dose and lung penetration was observed during volume-controlled ventilation as compared to pressure support ventilation.

The lung anatomy and the potential postoperative concerns (retention of bronchial secretions, minor atelectasis) more likely explain this high variability [35]. The relatively small variability of the nebulizer output (5 %) [6] and images quantification performed by two blind clinicians ensured the accuracy and the reproducibility of the analysis.

The aerosol distribution between both lungs was also variable among patients although a majority presented a higher aerosol deposition in the right lung. Two studies also found a higher aerosol delivery in the right lung of intubated patients following open-heart surgery [14, 16]. Our patients had healthy lungs unaffected by the surgical procedure. Repartition of the ventilation in the right/left lung depicted by the ^81m^Kr ventilation scan was close to 55/45 % indicating a normally distributed ventilation volume [36]. The increased right lung deposition could be explained by this physiological increased right lung ventilation [37]. The fact that the patient’s head was turned right for all patients may also explain the predominant right lung deposition because of an uncontrolled twist of the endotracheal tube. This had no effect on a volume tracer such as ^81m^Kr, but lead to more proximal bronchus deposition on the right side. As shown in Fig. 3b, c, this effect is rather versatile.

High aerosol impaction on the endotracheal tube and the trachea (20–27 % of the nominal dose) was measured with both modes. Larger particles which were generated at the inlet of the endotracheal tube directly impacted the lumen of the endotracheal tube. Higher impaction during pressure support is explained by the higher inspiratory flow rate inherent to this ventilation mode. Previous scintigraphic studies reported a comparable aerosol deposition within the ventilator circuit around 30 % [13, 14, 16]. However, we found a majority of aerosolized particles deposited on the proximal pieces of the ventilator circuit (proximal flow sensor, the catheter mount and the nebulizer T-piece).The expiratory filter captured around 20 % of aerosolized particles. Our nebulizer generated particles proximally during the entire breathing cycle. Particles were captured on proximal pieces of the ventilator circuit or directly lost on the expiratory limb during the long expiratory time measured in this study [7, 38].

Our study has several limitations. First, extrapolation of our results is limited due to the nebulization performed in patients with healthy lungs, using a radiotracer instead of a relevant drug, using a minimal mandatory sedation during pressure support ventilation and a ventilator circuit without heated and humidifier system. However, the aim of the study was to focus on the effect of mechanical ventilation on nebulization efficiency and not on its clinical benefit. Second, the continuous nebulizer was connected to the endotracheal tube. At the beginning of the study, the distal position was not known as the optimal position to reduce aerosol loss considering the presence of the proximal flow sensor on the ventilator circuit [7]. Further clinical study with nebulizer positioned at 30, 45 cm above the Y-piece or at the inspiratory outlet of the ventilator is required. Third, quantification was performed based on counts derived from a single anterior planar image instead of a geometric mean of anterior and posterior counts that could decrease the accuracy of the quantification [23]. Safety concern imposed to avoid patient’s transfer to the examination table and posterior images of the lungs could not be acquired due to bed structure; this bias, if of any relevance, was, however, constant for all patients in both groups.

In conclusion, volume-controlled ventilation was associated with higher aerosol delivery to the lungs as compared to pressure support ventilation with a vibrating-mesh nebulizer connected to the endotracheal tube in the specific conditions of the study. Lung deposition during pressure support ventilation was nonetheless substantial and should be further assessed using lighter sedation. Aerosol therapy is totally unpredictable in term to the location of aerosol deposition through the lungs. A high intersubject variability of lung doses, particles penetration and distribution between both lungs was demonstrated with the two ventilation modes. Before promoting volume control mode to administer an aerosol during invasive mechanical ventilation, we should assess the clinical benefit of the average 50 % differences in lung deposition in patients with a need for aerosol therapy such as inhaled antibiotics.

## Additional file

10.1186/s13613-016-0169-x Detailed description of particle size analysis and image correction for background, decay and attenuation for deposition analysis.
