# Supplementary material for: Aerosol delivery with two ventilation modes during mechanical ventilation: a randomized study
Source: Ann Intensive Care. 2016 Jul 22;6:73. doi: 10.1186/s13613-016-0169-x (PMC4958090; doi:10.1186/s13613-016-0169-x)
Supplement: Supplementary file 1 — 10.1186/s13613-016-0169-x Detailed description of particle size analysis and image correction for background, decay and attenuation for deposition analysis. [file 13613_2016_169_MOESM1_ESM.doc]

**Aerosol delivery with two ventilation modes during mechanical ventilation: a randomized study**

**Supplementary Material**

**Particle size analysis**

A test lung (5600i Dual Adult Training/Test Lung; Michigan Instrument Inc., Grand Rapids, MI) was connected to the ICU ventilator used in vivo through an 8- mm inner-diameter endotracheal tube. Leaks were prevented by inflating the endotracheal tube cuff in a 15-mm inner-diameter/22-mm outer-diameter adapter connected to the test lung. To keep a clinical perspective, the natural curve of the endotracheal tube was maintained. A slight inclination of the tube avoided drops deposition on the inner wall of tube lumen from reaching the filter media.

The throat of the impactor was replaced by a T-piece positioned between the endotracheal tube and the test lung to maintain an enclosed ventilator circuit. Plates of the impactor were previously coated with hydroxypropylmethylcellulose (0.5 mL HPMC /plate) to minimize particles bounce.

Nebulization was performed using the Aeroneb Solo® nebulizer for 5 minutes previously filled with 0.5 mCi of 99mTc-DTPA in 3mL of normal saline. A continuous flow of 28.3 L/min through the cascade impactor was maintained to attract aerosol particles that pass through the endotracheal tube to the cascade impactor. An opposite flow rate of 28.3 L/min inserted on the ventilator circuit from a medical air compressor (C250 Newport Medical, Medtronic, Minneapolis, MN) was used to maintain ventilator settings: tidal volume (V_T_) of 500 mL, respiratory rate (RR) 20 breaths/min, inspiratory time (T_i_) of 1 s, inspiratory time/breathing cycle time ratio (T_i_/T_TOT_) of 33%, positive end-expiratory pressure of 5 cmH_2_O.

**Images correction**

All images were corrected for background using mean pixel count of a region outside of the radiolabeled area. Total count rate in each ROI was calculated using this formula:


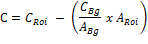


Each 99mTc images were also corrected for decay of the radioisotope using a time correction factor (TCF):


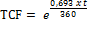


, where t was the time between the acquisition of the filled nebulizer and each image of 99mTc deposition. The resulting count rate corrected for background and attenuation was multiplied by this TCF.

Chest tissue attenuation of gamma rays should be corrected using an attenuation correction factor (ACF). The ACF for lungs was calculated using the flood-field source method (^57^Co flood-field source) for each patient. We used this formula:


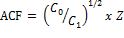


, where C_0_ and C_1_ are, respectively, the counts in a ROI from the flood-field source of ^57^Co without and with the subject present. Z equal to 0.95 for lungs corresponds to the conversion factor from a ^57^Co image with a lower gamma ray energy compared to a ^99m^Tc image.

We used a single ACF of 1.88 for the intra-patient part of the endotracheal tube based on recommendations from Pitcairn et al. (J Aerosol Medicine 1997:10; 187-98).
